# Projected Impact of Weight Gain During the COVID-19 Pandemic on the Future Burden of Cancer in Canada

**DOI:** 10.3389/fonc.2022.872765

**Published:** 2022-05-12

**Authors:** Rachel A. Murphy, Jaclyn Parks, Ryan Woods, Darren R. Brenner, Yibing Ruan, Parveen Bhatti

**Affiliations:** ^1^ School of Population and Public Health, University of British Columbia, Vancouver, BC, Canada; ^2^ Cancer Control Research, British Columbia (BC) Cancer, Vancouver, BC, Canada; ^3^ Data and Analytics, British Columbia (BC) Cancer, Vancouver, BC, Canada; ^4^ Department of Oncology, Cumming School of Medicine, University of Calgary, Calgary, AB, Canada

**Keywords:** obesity, cancer prevention, simulation, health behavior, pandemic

## Abstract

The COVID-19 pandemic and resulting public health measures have had significant impacts on daily life, including shifts in health behaviours which contribute to weight gain and may increase subsequent risk of chronic diseases such as cancer. Using OncoSim, a web-based microsimulation tool, we estimated the future burden of cancer in Canada by incorporating data on unintentional weight gain among adults during the first year of the COVID-19 pandemic. Population impact measures were estimated until 2042, assuming a 12-year latency period. We estimated 14,194 excess cancer cases and 5,324 excess cancer deaths by 2042 due to COVID-19 related weight gain. Particularly large impacts were estimated for endometrial and breast cancer among women, with 2,983 and 2,151 excess cases by 2042. For men, 1,700 excess colorectal cases and 1,188 excess kidney cancer cases were projected by 2042. Changes in health behavior during the COVID-19 pandemic are likely to have significant and long-lasting impacts on cancer burden. These projections highlight the immediate need for investment into the development and implementation of effective cancer prevention strategies.

## Introduction

In 2020, the coronavirus (COVID-19) pandemic and pandemic responses changed the daily behavior of billions of people around the world. In Canada, public health measures such as lockdown or quarantine, teleworking, school/childcare closures, and restricted access to or closure of recreational facilities, parks and playgrounds (1), had profound impacts on lifestyle. In a survey of nearly 10,000 Canadians, 74% reported that the pandemic impacted their eating habits, namely greater consumption of ‘unhealthy’ food (2). Nearly 25% of Canadians reported greater alcohol consumption (3), and physical activity levels have generally declined, with concurrent increases in time spent sedentary during the COVID-19 pandemic (4, 5). These changes have likely fueled increases in body weight; 42.3% of Canadians reported unintentional weight gain over the first year of the COVID-19 pandemic, with 39% reporting a weight gain of at least 11 pounds (lbs) (2).

Although it is unclear whether changes in health behaviors will persist with future waves of infection or waning of the COVID-19 pandemic, habit-formation principles suggest that health behaviors in response to environmental cues reach peak automaticity after ~two months (6). Findings from randomized-controlled trials have shown that small changes are not effective for weight loss among adults (7). Large, persistent shifts in health behaviors are needed, which suggests weight gained during the pandemic may linger. It is also possible that the “exposure period” of changes in health behavior is already sufficient to increase the risk of chronic disease.

Most of the scientific and clinical discourse which have sought to characterize the potential impact of the COVID-19 pandemic on cancer control have focused on delays in cancer screening and/or diagnosis. For example, early estimates of the impact of cancer screening program interruptions in Canada due to the COVID-19 pandemic projected a surge in breast and colorectal cancer cases diagnosed at advanced stages (8). However, given the strong evidence that health behaviors, particularly excess body weight, increase the risk of cancer (9), consideration of possible wider impacts of the COVID-19 pandemic such as shifts in risk factors that have the potential to impact the cancer control system in the future is warranted. In this microsimulation study, we aimed to estimate the possible long-term impacts of weight change during the COVID-19 pandemic on cancer incidence and mortality among Canadians.

## Materials and Methods

Impacts on cancer incidence and mortality were projected using OncoSim (version 3.4.0.3), a free, web-based microsimulation tool (10–12). OncoSim combines inputs such as demography, cancer risk factors, natural history of tumor development and progression to estimate impacts on cancer incidence and mortality relative to a baseline or counterfactual scenario (13). OncoSim models were developed using Canadian data whenever possible, and calibrated to match data from the Canadian Cancer registry and Canadian Vital Statistics. Simulations are run for one individual at a time and replicate the age, sex and all-cause mortality of the population in all provinces and territories in Canada. Model inputs can be modified to simulate the impact of counterfactual changes relative to baseline scenarios, where there is no change. Model outputs include age-standardized rates of cancer incidence and mortality, among other options. For each cancer type, the potential impact fraction (PIF) is calculated, which indicates the proportion of incident cancers that could be prevented if the risk factor in question (weight change) did not occur. Additional details of the simulation model, including validation and applications have been previously published (10, 11).

For this analysis, the simulated impacts of change in body mass index (BMI), which is categorized by sex and 5-year age groups, were modelled in OncoSim. Self-reported height and weight data from 96,237 individuals ages 20 and up in the most recent publicly available cycle (2017/2018) of the Canadian Community Health Survey (CCHS) were used to calculate baseline proportions for each BMI category (normal weight, overweight, obesity or morbid obesity) by age and sex (see Supplement) (14). Changes in weight were modeled using data from a survey (Agri-Foods) of 10,000 Canadian adults in which 15.6% unintentionally lost weight, 42.1% maintained their weight, and 42.3% unintentionally gained weight from March 2020 to April 2021 (2). Participants that gained weight were asked how much weight they gained in pre-specified categories (e.g. 1 to 5 pounds, 6 to 10 pounds etc.); however, amounts of weight loss were not queried. The amount of weight gain was randomly assigned from the midpoint of each weight gain category (e.g. 3 lbs for 1-5 lbs category) in the proportions reported in the survey to body weights reported for each participant in the 2017/2018 CCHS data. Weight loss was modeled using a similar approach, where 15.6% of CCHS participants were randomly assigned a value of weight loss from a normal distribution with a mean of 5.27 lbs, based on weight loss reported in a study of adults and weight management during the COVID-19 pandemic (15). BMI was then recalculated, and the CCHS baseline and revised BMI distributions were entered into OncoSim for each age group, by sex (overall BMI categorization found in **Table 1**). Parameters were set such that the change in BMI occurred between 2020 and 2021, with the revised BMI distribution held constant after 2021. Age group specific incident cancer cases and deaths projected under the baseline scenario were used to infer incident cancers among age groups under the counterfactual scenario (**Supplementary Tables S2, S3**).

**Table 1:** The distribution of BMI before and after weight gain among participants in the CCHS assuming 42.3% of population gained weight and 15.6% lost weight.

BMI Categorization	Normal or underweight, %(n)	Overweight,%(n)	Obese I, %(n)	Obese II, %(n)
Before weight change	41.2 (39,616)	35.2 (33,879)	15.8 (15,166)	7.9 (7,576)
After weight change	35.7 (34,373)	36.6 (35,219)	18.1 (17,411)	9.6 (9,228)

Percentages are reported to the nearest one decimal place for those of age 20 and up on the 2017/18 Canadian Community Health Survey dataset (n=96,237). Normal or underweight: BMI <25kg/m^2^, overweight: BMI 25-29.9kg/m^2^, Obese I: BMI 30-34.9kg/m^2^, and Obese II: BMI ≥ 35kg/m^2^.

Two alternative models of weight change were considered. First, the counterfactual scenario was altered to address possible age-related changes in body weight that are estimated to be ~1 to 2 lbs a year for American adults (16). OncoSim is unable to model dynamic changes in weight, and as such, a value from a normal distribution with a mean of 1.65 pounds was added to the body weight of all adults between the ages of 20-65, reflecting a change in BMI from 2020 to 2021. BMI was then recalculated by applying this weight change to the weight reported in the 2017/2018 CCHS and the revised BMI was entered into OncoSim. Second, the potential for weight gain to be transient was modelled using a ‘best case’ scenario where 20% of those who were categorized in the weight gain group in the main model were reallocated as weight maintainers. Findings from the primary simulation of weight change where the baseline assumed no change in weight were then compared to the findings from the additional analyses. For all models, the OncoSim default 12-year cancer latency period was applied (**Figure S1**, see Supplement).

Analyses of resulting data were performed using R version 3.5.1. This study was exempt from the Institutional Review Board at the University of British Columbia as it used publicly available data.

## Results

The baseline (pre-COVID-19) prevalence of normal or underweight (BMI<25.0 kg/m^2^), overweight (BMI 25.0-29.9 kg/m^2^), obesity (BMI 30.0-34.9 kg/m^2^) and morbid obesity (BMI ≥35.0 kg/m^2^) among Canadians was 41%, 35%, 16%, and 8% respectively. Of the estimated 42.3% who unintentionally gained weight, the mean weight gain was 11.3 lbs (inter-quartile range=5.0 lbs, minimum=3.0 lbs, maximum=51.0 lbs) during the first year of the COVID-19 pandemic in our simulated data. The mean weight loss for the 15.6% of the population who lost weight was 5.27 lbs (interquartile range=1.34 lbs, minimum=2.68 lbs, maximum=7.66 lbs). After accounting for weight changes, the proportions of those who were normal or underweight, overweight, obese and morbidly obese were 36%, 37%, 18%, and 10%, respectively (**Table 1**).

In the secondary model, the proportions of those normal or underweight, overweight, obese and morbidly obese after one year of a usual weight change were estimated to be 39%, 37%, 16% and 8%, respectively.

### Impact on Cancer Incidence

**Figure 1:**
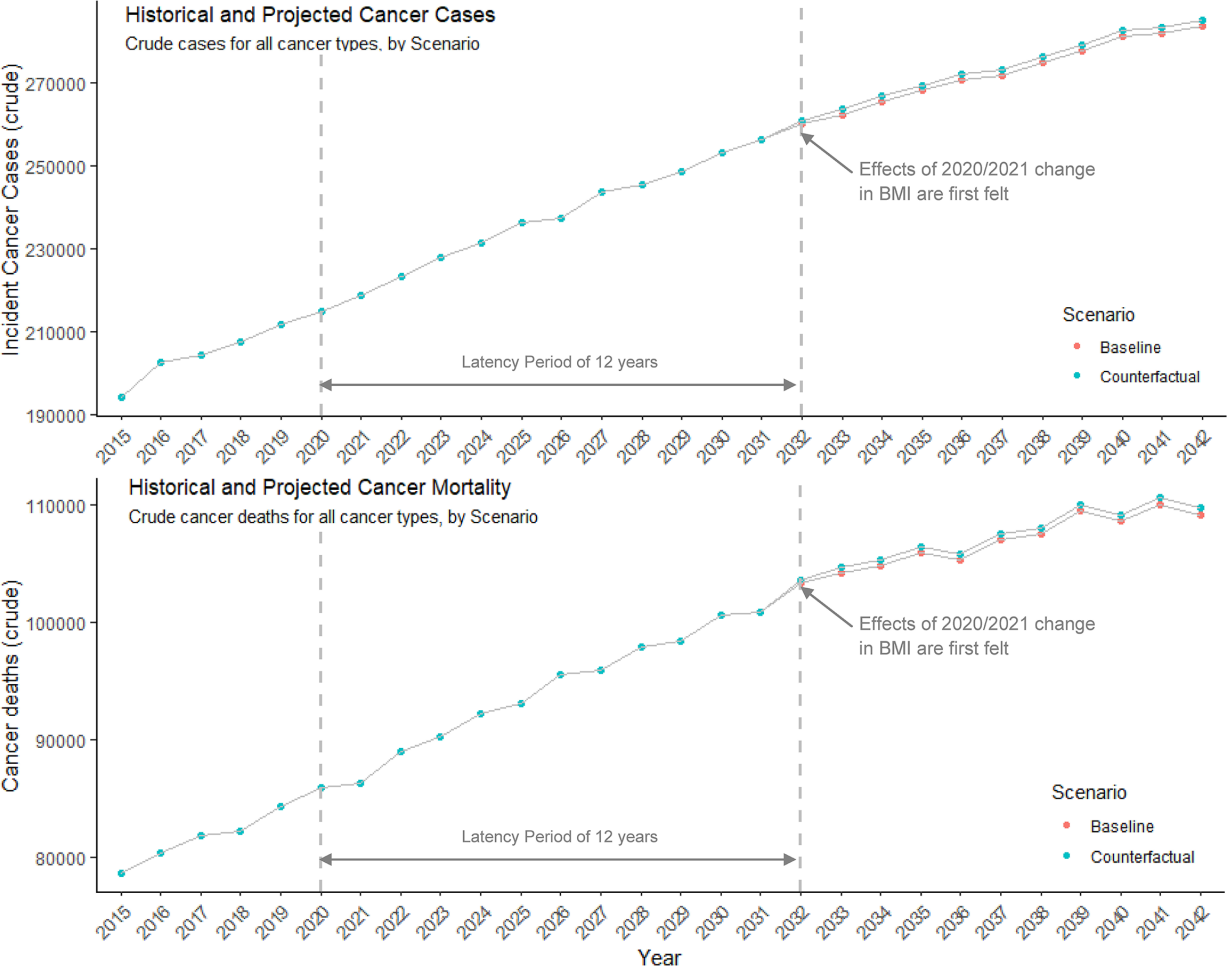
Historical and projected incident cancer cases and deaths for all cancer types. The number of new cancer cases and cancer deaths each year under baseline assumptions of cancer burden trajectories, and a counterfactual scenario where there are anticipated to be excess burdens attributed to pandemic-related changes in BMI. Effects of the pandemic-related onset and sustained BMI increase in 2020/2021 are not seen until 2032, due to the assumed 12-year latency period between excess body weight as a risk factor and the onset of cancer.

**Figure 2:**
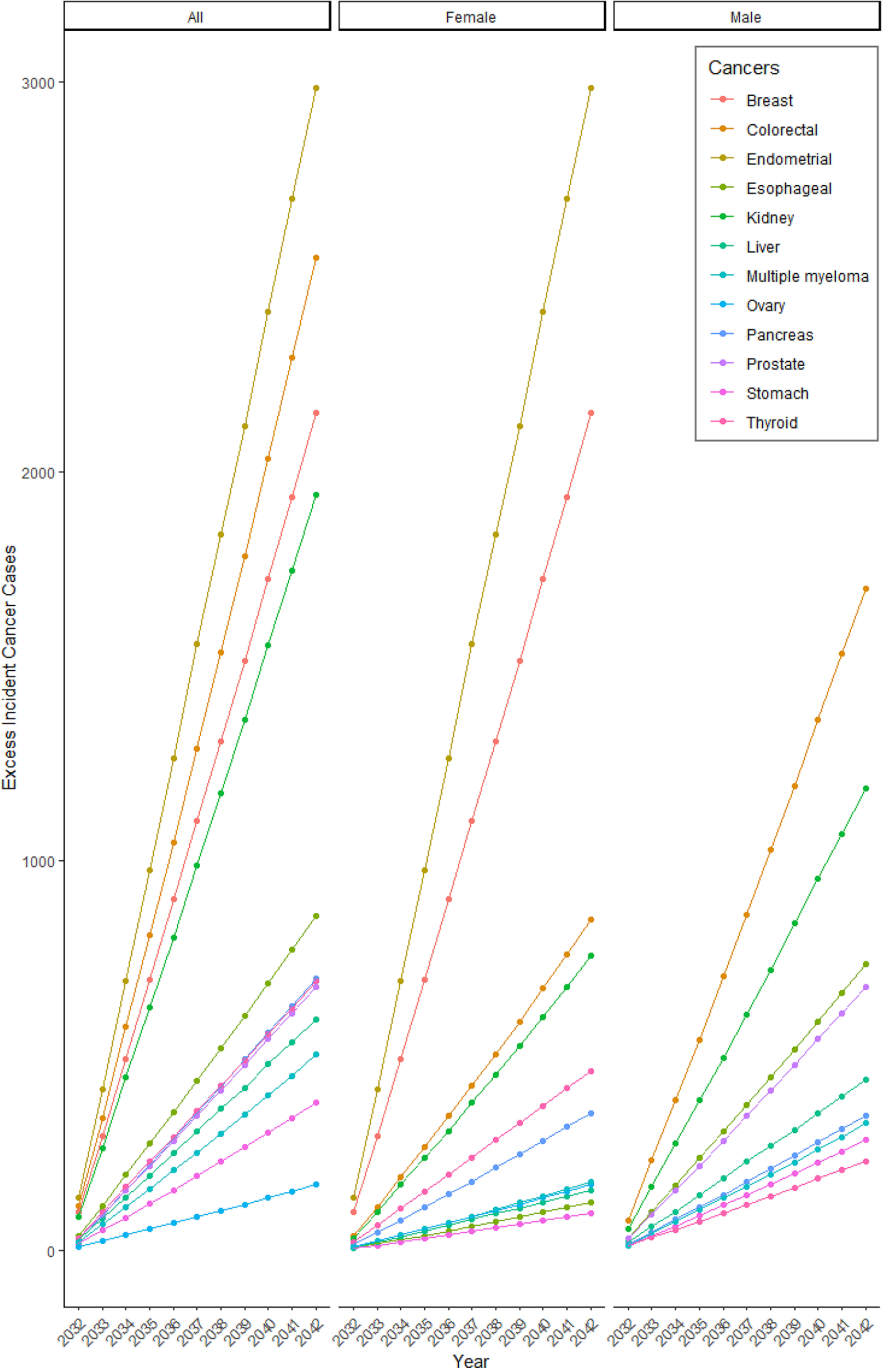
Projected cumulative excess incident cancer cases due to changes in BMI in 2020/21. The number of cumulative excess incident cancer cases projected each year due to excess burden attributed to pandemic-related changes in BMI in 2020/21.

Our simulation models suggested that weight changes during the first year of the COVID-19 pandemic would result in an additional 640, 7,254, and 14,194 incident cancer cases in Canada by 2032, 2037, and 2042, respectively (**Table 2** and **Figure 1**). The additional cancer burden will disproportionately impact women (**Figure 2** and **Table 3**), with 8,273 of these 14,194 excess incident cases occurring in women and the largest overall projected increase in cases occurring for cancer of the endometrium (2,983 excess cases by 2042, PIF=3.43% (95% CI; 3.07%-3.80%)), and second largest increase for breast cancer (2,151 excess cases by 2042, PIF=0.63% (95% CI; 0.56%-0.70%)). Among men, 5,921 total excess cancer cases are projected by 2042, including 1,700 new colorectal cases, and 1,188 new kidney cancer cases (**Figure 2** and **Table 3**). The age groups expected to have the greatest additional burden vary by cancer type (**Supplementary Table S2**). For example, approximately one-third of endometrial cancer cases are expected to occur among women aged 60-70 years, while overall, 55% of excess incident cancers are expected to occur among those aged 65-85.

**Table 2:** Projected cumulative excess cancer incidence and mortality due to body weight change during the COVID-19 pandemic in 2020/21 assuming 42.3% of population gained weight and 15.6% lost weight.

Cancer site	Cumulative Excess Cancer Cases			Cumulative Excess Cancer Deaths		
	2032	2037	2042	2032	2037	2042
Esophageal	36	434	858	36	422	835
Stomach	17	190	380	11	121	239
Colorectal	114	1,287	2,548	41	475	934
Liver	28	307	594	21	233	445
Pancreas	31	353	699	31	349	691
Breast	99	1102	2,151	18	194	376
Endometrium	137	1,555	2,983	26	297	577
Ovary	8	86	169	5	56	110
Kidney	86	988	1,941	30	327	642
Prostate	30	345	677	5	57	113
Thyroid	32	357	631	1	17	33
Multiple myeloma	21	251	503	15	163	328
All	640	7,254	14,194	239	2,711	5,324

Cancer cases refers to incident cases of cancer, reported based on the most recent type of cancer diagnosed. Cancer deaths refers to mortalities where cancer is reported as the cause of death. Cases are reported to the nearest whole number and are presented as a cumulative value up to and including each year presented.

**Table 3:** Projected cumulative excess cancer incidence and mortality due to body weight change during the COVID-19 pandemic in 2020/21 by sex, assuming 42.3% of population gained weight and 15.6% lost weight.

Year	Cumulative Excess Cancer Cases						Cumulative Excess Cancer Deaths					
	Females			Males			Females			Males		
	2032	2037	2042	2032	2037	2042	2032	2037	2042	2032	2037	2042
**Cancer site**												
Esophageal	5	61	125	31	373	734	6	59	121	30	363	714
Stomach	4	48	97	13	142	283	3	33	66	5	88	173
Colorectal	37	424	848	76	863	1,700	14	159	316	28	316	618
Liver	7	80	154	21	227	440	6	76	147	15	157	298
Pancreas	15	177	352	12	175	347	15	175	348	15	173	342
Breast	98	1,102	2,151	–	–	–	18	194	376	–	–	–
Endometrium	137	1,555	2,983	–	–	–	26	297	577	–	–	–
Ovary	8	70	169	–	–	–	5	56	110	–	–	–
Kidney	31	381	756	55	607	1,188	10	114	231	20	212	411
Prostate	–	–	–	30	345	677	–	–	–	5	57	113
Thyroid	21	239	462	11	118	229	1	6	12	1	11	21
Multiple myeloma	8	87	176	13	164	327	5	58	118	10	105	211
All	373	4,242	8,273	266	3,013	5,921	108	1,228	2,423	131	1,483	2,901

Cancer cases refers to incident cases of cancer, reported based on the most recent type of cancer diagnosed. Cancer deaths refers to mortalities where cancer is reported as the cause of death. Cases are reported to the nearest whole number and are presented as a cumulative value up to and including each year presented.

In the secondary analysis of usual age-related weight change, an additional 145, 1,596, and 3,032 incident cancer cases were projected by 2032, 2037, and 2042, respectively. Of the 3,032 excess incident cases projected by 2,042, 1,679 are expected to occur in males and 1,353 in females. In the analysis of 33.8% gaining weight, an additional 492, 5,584 and 10,904 excess incident cases were projected by 2032, 2037 and 2042, respectively.

### Impact on Cancer Deaths

**Figure 3:**
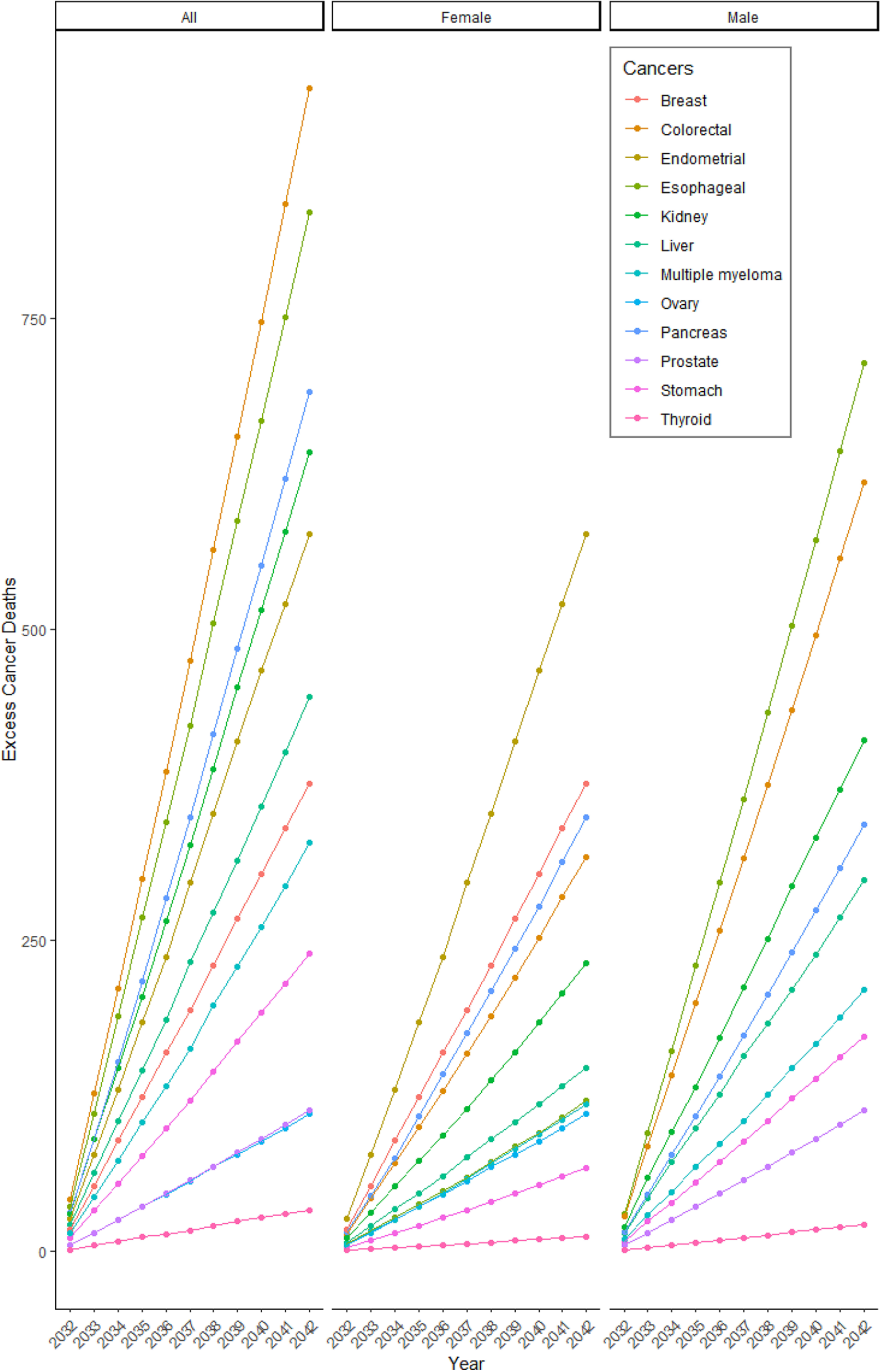
Projected cumulative excess cancer mortality due to changes in BMI in 2020/21. The number of cumulative excess cancer deaths projected each year due to excess burden attributed to pandemic-related changes in BMI in 2020/21.

Our simulation models projected 5,324 additional cancer deaths by 2042 due to weight gain during the first year of the COVID-19 pandemic (**Table 1** and **Figure 1**). The largest burden of deaths were projected for colorectal, esophageal and pancreatic cancer (N=934, N=835, and N=691 deaths, **Figure 3** and **Table 3**). Among women, the burden was projected to be particularly large for endometrial (N=577 deaths) and breast cancers (N=376 deaths). Among men, the largest burden was projected for esophageal (N=714 deaths) and colorectal cancers (N=618 deaths). The majority of the overall excess cancer mortality is expected to occur in ages 70-85 (49%), though there is variation by cancer type (**Supplementary Table S3**). For example, of the projected deaths in 2032-2042 due to prostate cancer, 93% will occur in those aged 60 and older while 55% of the projected deaths due to thyroid cancer will occur in those under 60 years of age.

In the secondary analysis of usual age-related weight change, there was projected to be an additional 56, 608, and 1,148 deaths due to cancer by 2032, 2037, and 2042, respectively. Of the 1,148 excess deaths by 2042, the majority (n=798) will be among men. In the analysis of 33.8% gaining weight, an additional 183, 2,079 and 4,072 excess cancer deaths were projected by 2032, 2037 and 2042 respectively.

## Discussion

Due largely to population growth and aging, the number of incident cancers diagnosed in Canada each year was previously estimated to increase from 96,440 in 2012 to 176,704 in 2042 (15). Our results suggest that changes in body weight during the COVID-19 pandemic may compound this surge with additional cancer cases, placing further burden on the healthcare system. The economic burden of cancer in Canada is already substantial at an estimated $7.5 billion in 2012, with an increase of $4.6 billion since 2005 (17). Our findings also highlight the need to consider the possible wider impacts of the COVID-19 pandemic beyond the more visible impact on healthcare utilization. The additional projected cancer deaths (5,324 by 2042) exceed those estimated due to a six-month interruption in cancer screening in Canada during the COVID-19 pandemic, which suggested an extra 250 cancer deaths by 2029 (8). Further, although the focus of our study was on body weight and cancer, the implications of changes in body weight and health behaviors that impact body weight are relevant to other chronic diseases beyond cancer. For example, it was recently projected that a conservative 10% reduction in leisure-time physical activity would result in 1,667 new cases of cardiovascular disease among Canadians over a 3-year period (18).

The objective of this simulation was to provoke thought about health behaviors during COVID-19, and potential long-term impacts on cancer control in the future. To do so, a number of assumptions were made, and the findings should be interpreted in that light. The estimates of cancer burden are sensitive to the representativeness of COVID-19 related weight change data, which have been scarce in Canada during the COVID-19 pandemic. We used data from the Agri-Foods survey (2) as it was the largest characterization (~10,000 Canadians) of weight change available at the time of our analysis. The prevalence of weight gain and amount of weight gained during the COVID-19 pandemic according to the Agri-Foods survey is similar to what has been reported by U.S. studies (19, 20). The Agri-Foods survey did not query the specific amount of weight loss, so a smaller U.S. study was referenced (15), which may have introduced error. The implications of unintentional weight loss with respect to cancer risk may be distinct from those of intentional weight loss, since the latter co-occurs with health promoting behaviors like exercise and healthy eating (21, 22), while the former co-occurs with adverse health factors/behaviors such as stress, smoking and alcohol consumption or certain chronic diseases (22, 23). As such, the estimated cases and deaths may be conservative.

The impacts of COVID-19 have been disproportionately experienced by those with lower SES and ethnic minorities (24, 25). We were unable to account for such disparities in our analysis since weight change data were not available at more granular levels (e.g. by ethnicity, socioeconomic status). The inability to account for natural age-related changes in body weight that equate to approximate gains of 1 -2 lbs per year until old age is a further limitation that may have skewed the counterfactual scenario, although this would also be relevant for those that reported weight change during the COVID-19 pandemic. Findings from our secondary analysis where a small increase in weight was applied to all adults aged 20-65y or the proportion gaining weight was reduced blunted the impacts of weight gain due to the COVID-19 pandemic on cancer incidence and mortality. However, one could argue that even a slight increase in cancer incidence and/or mortality is meaningful. Lastly, a key assumption of our models is that after the first year of the pandemic, body weight is maintained overtime as OncoSim is currently unable to simulate impacts of dynamic shifts in body weight or other cancer risk factors. Future iterations of the OncoSim platform may have increased capability to enable more nuanced simulations. While it is too soon to determine the long-term persistence of weight gain experienced during the COVID-19, a U.S. study reported that 33% of individuals who gained weight during COVID-19 lockdown periods continued to gain weight after the easing of lockdown measures, with 25% maintaining higher body weight (20).

The COVID-19 pandemic has necessitated a shift in public health priorities. A balance, however, may need to be struck, in the long-term, to mitigate the ongoing burden of non-communicable diseases, and the possible increases in cancer incidence and mortality resulting from impacts of the pandemic on health behaviors.

## Data Availability Statement

The raw data supporting the conclusions of this article will be made available by the authors, without undue reservation.

## Ethics Statement

Ethical review and approval was not required for the study on human participants in accordance with the local legislation and institutional requirements. The patients/participants provided their written informed consent to participate in this study.

## Author Contributions

RAM and PB conceived the idea. RAM, PB, and JP created the analytical plan with input from YR, DB, and RW. JP performed the analysis and compiled the results. RAM, PB, and JP wrote the manuscript. All authors provided critical feedback and contributed to the final manuscript.

## Funding

RAM’s time was funded by the Michael Smith Foundation for Health Research (grant #17644). OncoSim is led and supported by the Canadian Partnership Against Cancer, with model development by Statistics Canada, and is made possible through funding from Health Canada. The assumptions and calculations underlying the OncoSim simulation results were prepared by BC Cancer and the responsibility for the use and interpretation of these data and their reporting is entirely that of the author(s).

## Conflict of Interest

RAM is a consultant to Pharmavite LLC.

The remaining authors declare that the research was conducted in the absence of any commercial or financial relationships that could be construed as a potential conflict of interest.

## Publisher’s Note

All claims expressed in this article are solely those of the authors and do not necessarily represent those of their affiliated organizations, or those of the publisher, the editors and the reviewers. Any product that may be evaluated in this article, or claim that may be made by its manufacturer, is not guaranteed or endorsed by the publisher.
