# Supplementary material for: Projected Impact of Weight Gain During the COVID-19 Pandemic on the Future Burden of Cancer in Canada
Source: Front Oncol. 2022 May 12;12:872765. doi: 10.3389/fonc.2022.872765 (PMC9135177; doi:10.3389/fonc.2022.872765)
Supplement: Supplementary file 1 [file DataSheet_1.doc]

***Supplementary Material***

**Detailed Methods**

Canadian Community Health Survey (CCHS) data on height, weight and BMI data from the 2017/2018 cycle were grouped by age (based on 5-year categories in OncoSim) and sex. Of the data available for each sex and age range specification, 1,000 participants were randomly selected to serve as our baseline measure of the BMI distribution of the Canadian population in 2017/18. For example, there were 3,692 men between the ages of 50-54 with BMI information in the CCHS data. Of the 3,692, 1,000 were randomly selected to serve as the baseline population for men between the ages of 50-54. The BMI of participants in this sample of 1,000 50-54 year old men informed the baseline proportions for each BMI category that was subsequently entered into OncoSim.

The ‘counterfactual’ or modified proportions expected after pandemic-related unintentional weight change was determined using the AgriFoods survey that asked Canadian participants to report any change in their weight experienced March 2020-April 2021 (1). They reported that 42.3% of the population gained weight, 15.6% lost weight, and 42.1% maintained their weight from 2020-2021. These proportions were used to inform the counterfactual population scenario for each age and sex group. The amount of weight gain in 5-lb increments was also reported. For example, 23.5% gained between 1-5 lbs, 37.3% gained 6-10 lbs, and 17.9% gained 11-15 lbs. The midpoint of each 5-pound incremental weight gain category was randomly assigned to a dummy distribution of 1,000 records in the proportions reported by the AgriFoods study, with 423 of these participants being assigned a weight gain value. For example, a weight gain of 3 lbs was assigned to 99 participants (23.5% of 423), and a weight gain of 8 lbs was assigned to 158 participants (37.3% of 423). The AgriFoods survey did not ask about the amount of weight lost. As such, we applied a weight loss value from a normal distribution with a mean of 5.27 pounds based on the average weight loss reported by Borgatti et al (2).

The dummy dataset of 1,000 participants where 423 had a weight gain value, 156 a weight loss value, and 421 no change was matched with the randomly selected sample of 1,000 participants from each age group and sex from the Canadian Community Health Survey. Of the 1,000 participants randomly selected from each age and sex group (e.g. 50-54 year old males), 423 were randomly assigned a weight gain value, 156 were assigned a weight loss value, and the remaining 421 were not assigned any change in weight.

The post-pandemic distribution of BMI was calculated taking into account this positive, negative or no change in weight. The weight change value was divided by the baseline weight, as reported in the 2017/18 CCHS survey to produce a percent change. This percent change was applied to the baseline BMI to produce a modified BMI. The modified distribution of BMI for each age and sex group was assessed to determine the proportion of participants in each BMI category (i.e. normal weight, overweight, obese I, obese II) after a change in weight (if applicable). For example, the BMI distribution for 50-54 year old men changed from 29.4% with normal weight, 43.7 overweight, 18.5% class I obesity, and 8.4% class II obesity at baseline to 24.7%, 43.6%, 21.8%, and 9.9%, respectively. These proportions were entered into OncoSim as the counterfactual distribution for the ‘Excess Body Weight’ risk factor in OncoSim. It was specified that the change from baseline BMI to modified BMI proportions would take place between 2020-2021, with the revised BMI proportions completely in place by 2021. The same approach was taken to model potential impacts of transient weight gain where the proportion of participants characterized as weight gainers was reduced by 20%, and those participants were reallocated as weight maintainers.

For the analysis of usual age-related weight change, every person between the ages of 20-65 was randomly assigned a weight change value from a normal distribution with a mean of 1.63 pounds (min=-1.57, Median=1.66, max=4.39). This value was used to calculate the percent change in baseline weight, which was applied to their baseline BMI to produce a modified BMI. The distribution of BMI for participants in each age and sex group was assessed and the revised proportions were entered into OncoSim. The projections reflect the change in cancer burden as a result of the change in BMI distribution between 2020-2021 if weight had changed under normal non-pandemic circumstances.

**References**

1) Charlebois S. COVID‑19 WELL BEING. Agri-Food Analytics Lab. Cited 2021 Sep 1. Available from: https://cdn.dal.ca/content/dam/dalhousie/pdf/sites/agri-food/COVID%20Well%20Being%20(April%2018%202021)%20EN.pdf

2) Borgatti AC, Schneider-Worthington CR, Stager LM, Krantz OM, Davis AL, Blevins M, Howell CR, Dutton GR. The COVID-19 pandemic and weight management: Effective behaviors and pandemic-specific risk factors.(2021). Obesity Research and Clinical Practice. 15 (5);518-521.

**Table S1.** Potential impact fractions assuming 42.3% of population gained weight and 15.6% lost weight

| **Cancer site** | **Potential Impact Fraction (%) Females** | | **Potential Impact Fraction (%) Males** | |
| --- | --- | --- | --- | --- |
|  | **PIF** | **95% CI** | **PIF** | **95% CI** |
| Esophageal | 1.48 | 1.32-1.63 | 2.70 | 2.41-2.99 |
| Stomach | 0.45 | 0.40-0.50 | 0.75 | 0.67-0.83 |
| Colorectal | 0.59 | 0.53-0.65 | 0.98 | 0.87-1.08 |
| Liver | 1.71 | 1.53-1.89 | 1.61 | 1.44-1.78 |
| Pancreas | 0.85 | 0.76-0.94 | 0.80 | 0.71-0.88 |
| Breast | 0.63 | 0.56-0.70 | - | - |
| Endometrial | 3.43 | 3.07-3.80 | - | - |
| Ovary | 0.39 | 0.35-0.43 | - | - |
| Kidney | 2.11 | 1.88-2.34 | 2.02 | 1.80-2.24 |
| Prostate | - | - | 0.16 | 0.14-0.17 |
| Thyroid | 0.67 | 0.60-0.74 | 1.07 | 0.96-1.19 |
| Multiple myeloma | 0.84 | 0.75-0.93 | 1.22 | 1.09-1.35 |

The mean potential impact fraction percentage is displayed for the average value from 2032-2042. The 95% confidence interval is also reported to the nearest second decimal place. PIF is the fraction of disease incidence that could be prevented under a counterfactual scenario: PIF = (I – I*)/I x100%, in which I is the disease incidence at base scenario and I* is the disease incidence at the counterfactual scenario. Where excess body fat is not known to affect the risk of a particular cancer, there is no PIF generated. OncoSim only reports breast cancers and associated risks in females.

**Figure S1.**
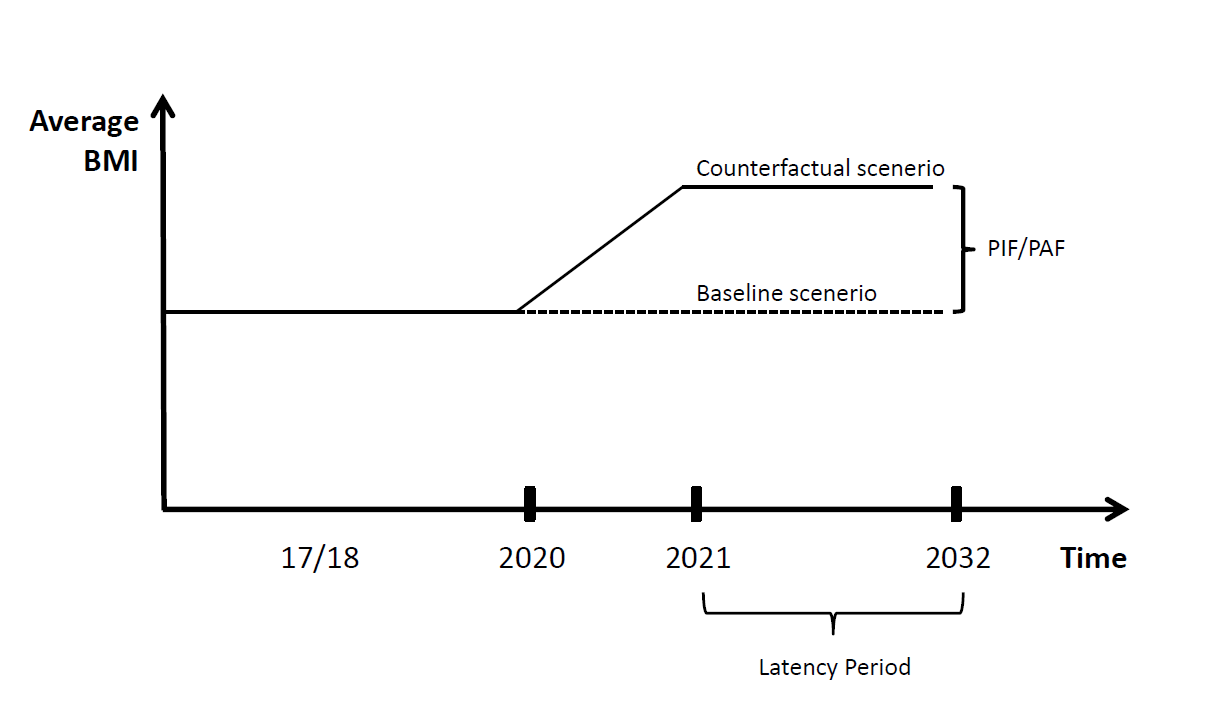
 Visual representation of the theoretical framework for running simulation models in OncoSim

For this analysis, parameters within Oncosim specified that the change in weight from the baseline distribution (based on the 2017/18 CCHS) to the counterfactual distribution (post-pandemic weight gain) occurred between the years 2020 and 2021. A latency period of 12 years was selected as the default, meaning that any change occurring in 2020 would take 12 years to materialize as a difference in cancer burden. The model was run on the OncoSim platform using a population of 32,000,000 cases to ensure adequate reliability in the produced results.

**Table S2.** Projected 2032-2042 cancer incidence distributed by age group for the main analysis

| Cancer site | Age group | | | | | | | | | | | | | | |
| --- | --- | --- | --- | --- | --- | --- | --- | --- | --- | --- | --- | --- | --- | --- | --- |
|  | 20-25 | 25-30 | 30-35 | 35-40 | 40-45 | 45-50 | 50-55 | 55-60 | 60-65 | 65-70 | 70-75 | 75-80 | 80-85 | 85-90 | 90+ |
| Esophageal | 0.02 | 0.04 | 0.17 | 0.32 | 0.77 | 1.95 | 4.08 | 7.44 | 9.72 | 13.66 | 16.19 | 16.84 | 14.17 | 8.84 | 5.80 |
| Stomach | 0.05 | 0.20 | 0.44 | 0.75 | 1.36 | 2.70 | 3.38 | 5.23 | 7.08 | 10.28 | 14.79 | 17.82 | 16.84 | 11.78 | 7.28 |
| Colorectal | 0.11 | 0.28 | 0.58 | 1.07 | 1.85 | 2.78 | 4.74 | 5.44 | 7.98 | 9.85 | 13.75 | 14.18 | 15.71 | 12.05 | 9.62 |
| Liver | 0.07 | 0.13 | 0.20 | 0.42 | 0.95 | 2.61 | 5.78 | 10.56 | 10.34 | 12.16 | 15.50 | 16.92 | 13.49 | 6.12 | 4.06 |
| Pancreas | 0.03 | 0.09 | 0.15 | 0.45 | 0.90 | 1.75 | 3.61 | 5.18 | 8.28 | 11.04 | 15.61 | 18.68 | 15.83 | 10.77 | 7.56 |
| Breast | 0.09 | 0.29 | 0.82 | 2.05 | 4.62 | 6.98 | 11.30 | 10.07 | 13.16 | 11.71 | 13.58 | 8.84 | 7.71 | 5.19 | 3.58 |
| Endometrial | 0.03 | 0.17 | 0.44 | 1.13 | 2.09 | 4.38 | 8.40 | 13.63 | 15.74 | 15.42 | 12.94 | 10.92 | 7.41 | 4.19 | 3.08 |
| Ovary | 0.59 | 0.79 | 1.07 | 1.66 | 3.78 | 5.32 | 7.65 | 8.09 | 9.53 | 10.90 | 12.66 | 13.52 | 11.27 | 7.23 | 5.43 |
| Kidney | 0.09 | 0.30 | 0.51 | 1.26 | 2.41 | 4.08 | 5.92 | 8.53 | 10.40 | 12.74 | 15.20 | 14.91 | 11.44 | 7.13 | 4.36 |
| Prostate | 0.00 | 0.00 | 0.00 | 0.02 | 0.27 | 1.39 | 4.36 | 8.89 | 14.04 | 19.16 | 17.83 | 14.27 | 9.55 | 6.45 | 3.78 |
| Thyroid | 1.74 | 3.62 | 5.96 | 8.86 | 10.48 | 10.81 | 11.03 | 10.13 | 9.54 | 8.84 | 7.62 | 5.77 | 2.60 | 1.10 | 0.71 |
| Multiple myeloma | 0.02 | 0.02 | 0.07 | 0.29 | 0.90 | 1.92 | 3.44 | 5.40 | 8.19 | 11.60 | 15.55 | 18.68 | 16.68 | 10.45 | 6.78 |
| All | 0.31 | 0.51 | 0.77 | 1.26 | 2.11 | 3.27 | 5.30 | 6.98 | 9.58 | 12.04 | 14.66 | 15.05 | 13.08 | 8.79 | 5.69 |

Values represent the proportion of each cancer case projected to occur from age 20 and older in each age group as a percent value to the nearest second decimal place. Due to rounding, the total row may not sum to 100%. Estimates for the cancer types linked to excess body fat are presented here. Estimates are based on the average annual projected cases due to cancer from 2032-2042 under the assumptions of a baseline weight distribution in the population. The baseline weight distribution is based on the BMI values reported in the 2017/2018 Canadian Community Health Survey.

**Table S3**. Projected 2032-2042 cancer mortality distributed by age group for the main analysis

| Cancer site | Age group | | | | | | | | | | | | | | |
| --- | --- | --- | --- | --- | --- | --- | --- | --- | --- | --- | --- | --- | --- | --- | --- |
|  | 20-25 | 25-30 | 30-35 | 35-40 | 40-45 | 45-50 | 50-55 | 55-60 | 60-65 | 65-70 | 70-75 | 75-80 | 80-85 | 85-90 | 90+ |
| Esophageal | 0.02 | 0.04 | 0.17 | 0.34 | 0.68 | 1.92 | 3.84 | 7.31 | 9.72 | 13.64 | 16.31 | 17.00 | 13.98 | 9.18 | 5.83 |
| Stomach | 0.04 | 0.17 | 0.44 | 0.76 | 1.26 | 2.52 | 3.30 | 4.92 | 6.75 | 10.08 | 14.43 | 17.38 | 17.54 | 12.07 | 8.34 |
| Colorectal | 0.07 | 0.22 | 0.49 | 0.96 | 1.84 | 2.71 | 3.87 | 4.70 | 6.57 | 8.88 | 12.21 | 14.76 | 16.70 | 14.11 | 11.90 |
| Liver | 0.08 | 0.16 | 0.21 | 0.38 | 0.84 | 2.10 | 4.61 | 8.79 | 9.84 | 12.29 | 15.98 | 17.39 | 14.18 | 7.68 | 4.80 |
| Pancreas | 0.04 | 0.09 | 0.13 | 0.45 | 0.89 | 1.69 | 3.58 | 5.15 | 8.18 | 11.06 | 15.51 | 18.63 | 15.95 | 10.86 | 7.69 |
| Breast | 0.02 | 0.09 | 0.33 | 0.81 | 1.84 | 3.73 | 6.73 | 7.79 | 8.86 | 10.59 | 13.50 | 14.97 | 13.81 | 9.55 | 7.36 |
| Endometrial | 0.03 | 0.05 | 0.34 | 0.80 | 1.51 | 3.46 | 5.52 | 9.95 | 13.36 | 15.04 | 15.00 | 13.73 | 10.73 | 5.82 | 4.63 |
| Ovary | 0.50 | 0.77 | 1.04 | 1.60 | 2.63 | 4.69 | 6.73 | 7.87 | 9.45 | 11.03 | 12.91 | 13.71 | 11.78 | 8.34 | 6.52 |
| Kidney | 0.15 | 0.27 | 0.52 | 0.99 | 1.90 | 3.51 | 4.97 | 8.07 | 9.52 | 11.83 | 15.39 | 15.73 | 12.86 | 8.39 | 5.17 |
| Prostate | 0.00 | 0.00 | 0.00 | 0.01 | 0.12 | 0.54 | 2.02 | 4.55 | 9.11 | 14.17 | 18.69 | 19.03 | 15.49 | 9.65 | 6.62 |
| Thyroid | 1.75 | 2.26 | 5.39 | 6.92 | 7.50 | 9.76 | 10.34 | 10.56 | 10.56 | 10.34 | 7.87 | 9.47 | 3.28 | 2.40 | 0.66 |
| Multiple myeloma | 0.01 | 0.01 | 0.06 | 0.20 | 0.69 | 1.57 | 2.59 | 4.37 | 7.28 | 10.32 | 15.18 | 18.76 | 17.80 | 12.30 | 8.88 |
| All | 0.22 | 0.30 | 0.46 | 0.74 | 1.26 | 2.16 | 3.55 | 5.17 | 7.25 | 10.27 | 14.97 | 17.79 | 16.58 | 11.22 | 7.42 |

Values represent the proportion of each cancer death projected to occur from age 20 and older in each age group as a percent value to the nearest second decimal place. Due to rounding, the total row may not sum to 100%. Estimates for the cancer types linked to excess body fat are presented here. Estimates are based on the average annual projected deaths due to cancer from 2032-2042 under the assumptions of a baseline weight distribution in the population. The baseline weight distribution is based on the BMI values reported in the 2017/2018 Canadian Community Health Survey.

**Table S4**. Projected cumulative excess cancer incidence and mortality costs due to body weight change during the COVID-19 pandemic in 2020/21 for the model assuming 33.8% of population gained weight and 15.6% lost weight

| **Cancer site** | **Cumulative Excess Cancer Cases** | | | **Cumulative Excess Cancer Deaths** | | |
| --- | --- | --- | --- | --- | --- | --- |
|  | **2032** | **2037** | **2042** | **2032** | **2037** | **2042** |
| Esophageal | 28 | 335 | 658 | 28 | 326 | 640 |
| Stomach | 13 | 145 | 289 | 8 | 92 | 182 |
| Colorectal | 87 | 980 | 1,935 | 32 | 262 | 710 |
| Liver | 21 | 235 | 453 | 16 | 178 | 340 |
| Pancreas | 24 | 268 | 530 | 23 | 265 | 524 |
| Breast | 75 | 854 | 1,666 | 14 | 151 | 292 |
| Endometrium | 106 | 1,202 | 2,302 | 20 | 229 | 445 |
| Ovary | 6 | 66 | 130 | 4 | 43 | 85 |
| Kidney | 66 | 760 | 1,491 | 23 | 252 | 493 |
| Prostate | 23 | 264 | 519 | 4 | 44 | 87 |
| Thyroid | 25 | 283 | 548 | 1 | 13 | 26 |
| Multiple myeloma | 16 | 192 | 383 | 11 | 124 | 250 |
| All | 492 | 5,584 | 10,904 | 183 | 2,079 | 4,072 |

Cancer cases refers to incident cases of cancer, reported based on the most recent type of cancer diagnosed. Cancer deaths refers to mortalities where cancer is reported as the cause of death. Cases are reported to the nearest whole number.
